# MSC‐Derived Secretome and Exosomes in Dermatology: Mechanisms, Therapeutic Opportunities, and Scientific Challenges—A Narrative Review

**DOI:** 10.1111/ijd.17982

**Published:** 2025-08-01

**Authors:** Marcela da Costa Pereira Cestari, Reinaldo Falavigna Tovo, Daniela Franco Bueno

**Affiliations:** ^1^ Instituto de Ensino e Pesquisa Hospital Sírio‐Libanês São Paulo São Paulo Brazil; ^2^ Pontifícia Universidade Católica de São Paulo (PUC‐SP), Campus Sorocaba Sorocaba São Paulo Brazil; ^3^ Faculdade Israelita de Ciências da Saúde Albert Einstein São Paulo São Paulo Brazil

**Keywords:** dermatoses, exosomes, extracellular vesicles, mesenchymal stem cell‐derived exosomes, mesenchymal stromal cells, secretome, skin diseases

## Abstract

Mesenchymal stromal cells (MSCs) exert their effects primarily through paracrine signaling via soluble factors and extracellular vesicles (EVs), especially exosomes. These acellular components offer regenerative and immunomodulatory benefits with fewer safety and logistical constraints than cell‐based therapies. This study aims to review the composition, mechanisms of action, and dermatologic applications of MSC‐derived secretomes and exosomes, including engineered and primed variants, and to discuss translational barriers and safety considerations. A structured literature search was conducted using PubMed and Embase. Studies on molecular content, preclinical and clinical data, engineered EVs, oncologic safety, and regulatory aspects of MSC‐derived products in dermatology were included. The MSC secretome includes cytokines, chemokines, growth factors, lipids, and regulatory RNAs that modulate inflammation, promote repair, and support skin homeostasis. Exosomes—particularly those from primed or engineered MSCs—play a key role via targeted microRNA delivery. Preclinical data support efficacy in atopic dermatitis, psoriasis, alopecia areata, vitiligo, chronic ulcers, and photoaging. Pilot clinical trials show promising safety and feasibility for topical or intradermal use. However, product heterogeneity, unclear dosing, long‐term oncologic safety, and regulatory challenges persist. MSC‐derived secretome and exosomes—especially those from primed or engineered MSCs—offer a promising acellular platform for dermatologic therapy. Clinical translation requires standardization, mechanistic validation, and rigorous safety evaluation through well‐designed trials.

## 1 Introduction

Mesenchymal stromal cells (MSCs) have gained attention in regenerative medicine due to their immunomodulatory and reparative effects, primarily mediated through paracrine signaling. Their secretome—comprising bioactive molecules—includes extracellular vesicles, particularly exosomes: nanosized, membrane‐bound particles that deliver proteins, lipids, and nucleic acids to regulate intercellular communication and tissue homeostasis [1, 2, 3]. This acellular strategy offers advantages over live‐cell therapies, including lower immunogenicity, easier storage and handling, and a favorable safety profile, and has been explored across specialties like neurology, cardiology, and orthopedics [4, 5].

These features are especially relevant in dermatology, where chronic inflammatory dermatoses, autoimmune disorders, non‐healing ulcers, and photoaging demand targeted, sustained immune and regenerative modulation. Conditions such as atopic dermatitis (AD), psoriasis, vitiligo, alopecia areata, and chronic wounds remain challenging—especially in refractory cases—and carry a significant psychosocial and economic burden [6, 7, 8, 9, 10, 11, 12]. Over the past decade, MSC‐based products have shown potential in dermatology. Preclinical studies indicate that MSC‐derived secretome and exosomes enhance skin barrier function, modulate inflammation, promote angiogenesis, and activate keratinocytes and fibroblasts [13, 14, 15, 16, 17, 18, 19]. Their suitability for topical or intradermal delivery further supports their use in skin therapies by allowing localized action with minimal systemic exposure [4, 20, 21, 22, 23]. These benefits have driven academic and commercial interest, leading to a proliferation of exosome‐based products for dermatologic and cosmetic use.

However, commercial enthusiasm has often outpaced scientific validation. Many marketed products claim efficacy and safety based primarily on preclinical or anecdotal data. The lack of standardized production methods, limited transparency about exosomal content, and scarce clinical evidence raise concerns about real‐world safety and effectiveness [4, 5, 24]. Recent reviews have highlighted these gaps, urging more rigorous scientific scrutiny [25, 26].

This narrative review critically examines the dermatologic applications of MSC‐derived secretome and exosomes, summarizing mechanisms of action, therapeutic potential, translational barriers, and areas requiring further research.

## 2 Methods

This narrative review is based on a non‐systematic literature search conducted from October 2024 to March 2025 in PubMed, Embase, Cochrane Central, Scopus, and LILACS. The search focused on studies evaluating mesenchymal stromal cell (MSC)‐derived secretome and extracellular vesicles—particularly exosomes—in dermatologic conditions.

Eligible sources included original preclinical and clinical research, systematic reviews, and relevant narrative reviews published in English between 2010 and 2025. Case reports, letters, and studies unrelated to dermatology were excluded.

Selection was based on relevance, scientific quality, and emphasis on the immunomodulatory, regenerative, and therapeutic roles of MSC‐derived products.

Artificial intelligence tools (ChatGPT‐4 by OpenAI) were used to support language editing and improve clarity during manuscript preparation. No content was generated autonomously; all data analysis and interpretation were performed by the authors. AI use complied with Wiley's Best Practices Guidelines on Research Integrity and Publishing Ethics.

## 3 Mesenchymal Stromal Cells—Mechanisms of Action

**FIGURE 1:**
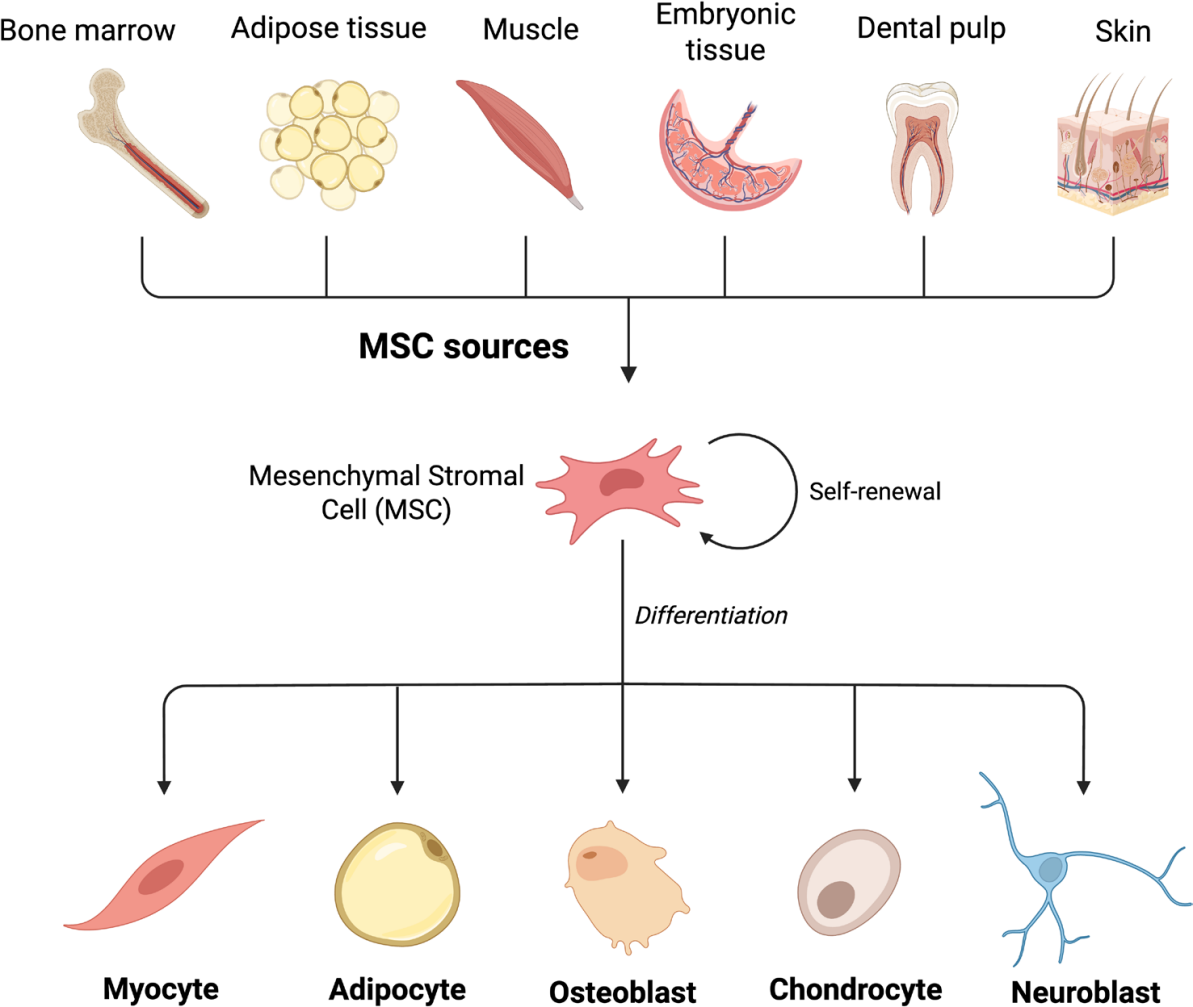
Mesenchymal stromal cells (MSCs) originate from multiple tissues and exhibit low immunogenicity, self‐renewal, and multilineage differentiation, making them suitable for regenerative therapies.

MSCs are multipotent, non‐hematopoietic cells capable of self‐renewal and differentiation into mesodermal lineages such as adipocytes, chondrocytes, and osteoblasts [27, 28, 29, 30]. Initially described by Friedenstein and later characterized by Caplan, they were first isolated from bone marrow and are now derived from various adult and fetal tissues, including adipose tissue, umbilical cord, placenta, dental pulp, and hair follicles [27, 31, 32, 33] (Figure 1).

According to the International Society for Cellular Therapy (ISCT), MSCs must adhere to plastic, express CD73, CD90, and CD105, lack hematopoietic markers, and differentiate into adipogenic, osteogenic, and chondrogenic lineages [28, 29]. Due to minimal MHC I and II expression, their low immunogenicity makes them suitable for allogeneic use [30, 34, 35].

While initially thought to act via direct engraftment, evidence now shows their primary mechanism is paracrine signaling [1, 2]. Seminal studies demonstrated that MSC‐conditioned medium could reproduce therapeutic effects in the absence of viable cells [36, 37, 38]. This shifted focus to the MSC secretome—comprising soluble factors and extracellular vesicles—as the main mediator of tissue repair and immune modulation [1, 2, 3].

MSCs actively respond to environmental cues by releasing cytokines, chemokines, and growth factors that support local regeneration, enhance resident cell function, and regulate immune responses [39]. This paracrine activity underpins growing interest in acellular MSC‐based therapies.

## 4 Composition and Functional Roles of the MSC‐Derived Secretome

The MSC‐derived secretome comprises a heterogeneous array of soluble factors and extracellular vesicles that act synergistically to modulate inflammation, promote angiogenesis, and support tissue remodeling [2, 3, 37, 40, 41]. The soluble fraction includes cytokines (e.g., interleukin [IL]‐10, IL‐6, tumor growth factor‐beta [TGF‐β], IL‐1Ra), chemokines (e.g., CCL2, CXCL12), and growth factors such as vascular endothelial growth factor (VEGF), hepatocyte growth factor (HGF), insulin‐like growth factor 1 (IGF‐1), and platelet‐derived growth factor (PDGF)—each of which plays a role in cutaneous regeneration by influencing immune cell recruitment, fibroblast activation, and re‐epithelialization [2, 16, 20, 42].

In the context of inflammatory dermatoses and chronic wounds, these mediators contribute to the downregulation of proinflammatory cytokines (e.g., tumor necrosis factor‐alpha [TNF‐α], IL‐1β), the promotion of macrophage polarization toward the anti‐inflammatory M2 phenotype, and the stimulation of keratinocyte proliferation and migration [16, 18, 19, 43]. This is particularly relevant in skin conditions characterized by persistent inflammation, impaired matrix remodeling, or defective angiogenesis.

Additional molecules such as prostaglandin E2 (PGE2), nitric oxide (NO), and indoleamine 2,3‐dioxygenase (IDO) further enhance immunosuppressive effects by modulating T cell responses and dendritic cell activity [42, 44]. These paracrine interactions are critical in restoring immune tolerance in diseases like atopic dermatitis and psoriasis and promoting wound resolution.

It is important to note that the MSC source influences secretome composition. Adipose tissue–derived MSCs (AD‐MSCs) secrete higher levels of angiogenic and anti‐inflammatory mediators, while bone marrow–derived MSCs (BM‐MSCs) exhibit greater hematopoietic and TGF‐β signaling activity [5, 44, 45, 46, 47, 48, 49]. Umbilical cord–derived MSCs (UC‐MSCs) and placental MSCs display intermediate profiles, often associated with stronger immunoregulatory capacity [44, 46, 49, 50].

These differences in paracrine profiles may account for variability in therapeutic responses and emphasize the need for source‐specific optimization of secretome‐based interventions in dermatology [16, 50, 51].

These bioactive components not only participate in tissue remodeling and repair but also play critical roles in regulating immune responses, as explored in the next section.

## 5 Immunomodulatory Effects on Innate and Adaptive Immunity

One of the most studied aspects of the MSC secretome is its ability to modulate both innate and adaptive immune responses. In innate immunity, MSC‐derived factors promote the polarization of macrophages from a pro‐inflammatory (M1) to an anti‐inflammatory (M2) phenotype, mainly through the action of IL‐10, PGE2, and TGF‐β [17, 40, 52, 53]. The secretome also downregulates mast cell degranulation, suppresses NK cell cytotoxicity, and modulates neutrophil and dendritic cell activity, contributing to a more controlled inflammatory response [17, 18, 42, 43, 53, 54, 55, 56].

In adaptive immunity, MSCs suppress T cell proliferation and shift helper T cell responses from Th1/Th17 to Th2 and Treg profiles. This effect is mediated by soluble factors such as IDO, PGE2, IL‐10, and TGF‐β, as well as by direct cell contact in some models [1, 17, 42]. Regulatory T cells (Tregs) are expanded, and pro‐inflammatory cytokines such as IFN‐γ and IL‐17 are reduced [1, 17, 42].

**FIGURE 2:**
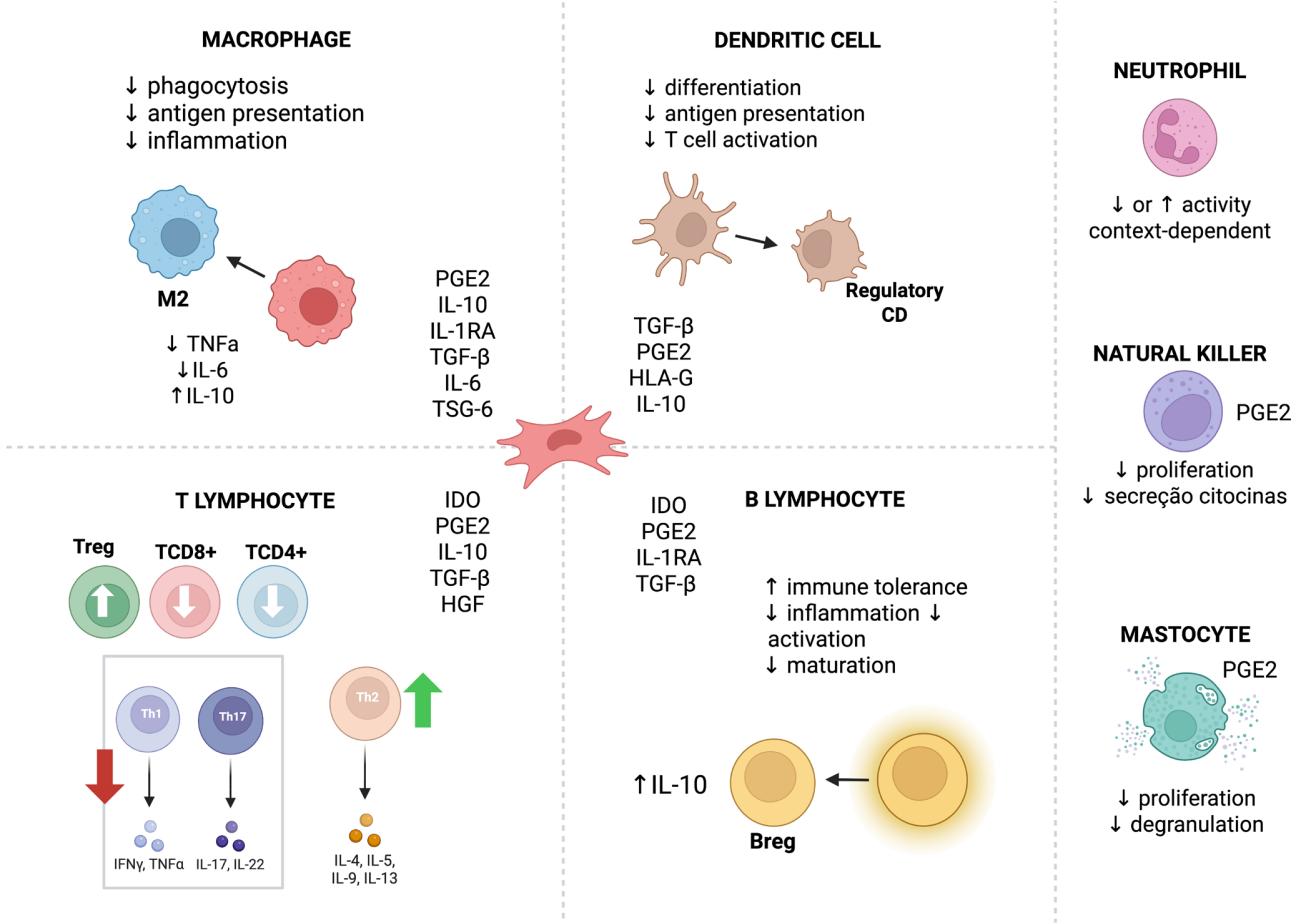
MSCs modulate innate and adaptive immunity by promoting M2 macrophage polarization, suppressing mast cells, NK cells, and proinflammatory Th1/Th17 responses, while enhancing Treg and Breg activity, supporting immune homeostasis and resolution of chronic inflammation.

Similarly, MSCs modulate B cell function by inhibiting their activation and promoting the generation of IL‐10‐producing regulatory B cells (Bregs) via TGF‐β, IL‐10, and PGE2 [42]. This broad immunomodulatory profile fosters an environment conducive to the resolution of chronic inflammation and supports tissue repair (Figure 2).

Overall, the MSC secretome plays a central role in controlling immune dysregulation while promoting regeneration, and this dual action forms the basis for its potential therapeutic use in dermatological diseases.

## 6 Extracellular Vesicles (EVs)

**FIGURE 3:**
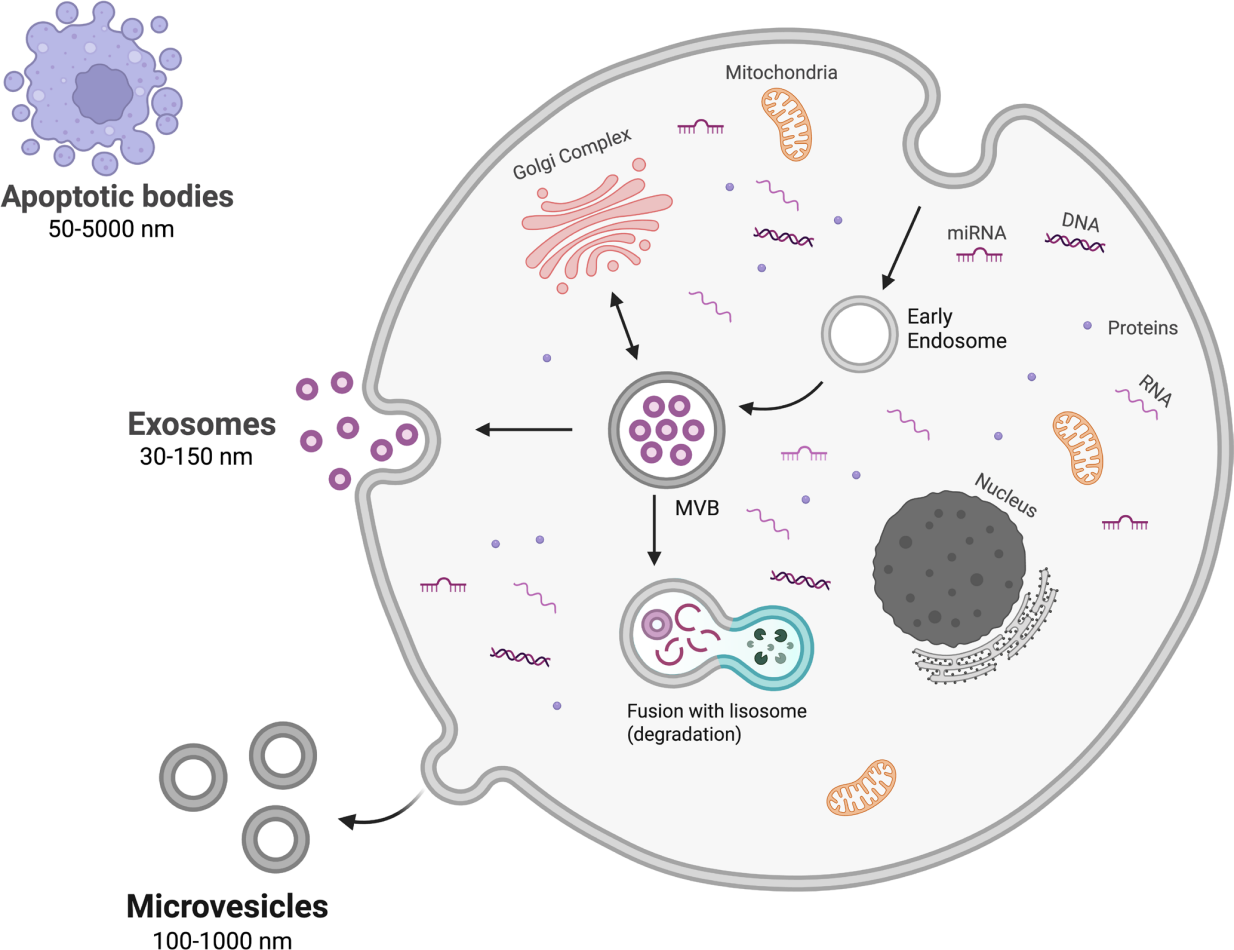
Exosome biogenesis: Exosomes form through the endosomal pathway and are released into the extracellular space upon multivesicular body fusion with the plasma membrane.

Extracellular vesicles (EVs) are membrane‐bound particles secreted by virtually all cell types, playing key roles in intercellular communication. MSC‐derived EVs are typically classified into apoptotic bodies, microvesicles, and exosomes, which differ in size, biogenesis, and function [57, 58, 59, 60, 61]. Exosomes, the smallest (30–150 nm), originate from the endosomal pathway and are released via fusion of multivesicular bodies with the plasma membrane [57, 59, 62] (Figure 3).

MSC‐derived exosomes carry a rich cargo of proteins, lipids, and nucleic acids—especially microRNAs (miRNAs)—and are considered major mediators of MSC paracrine activity [45, 63, 64, 65]. Their composition reflects the parent cell's activation state and varies with environmental stimuli such as hypoxia, inflammation, or mechanical stress [45, 64, 66, 67, 68].

**FIGURE 4:**
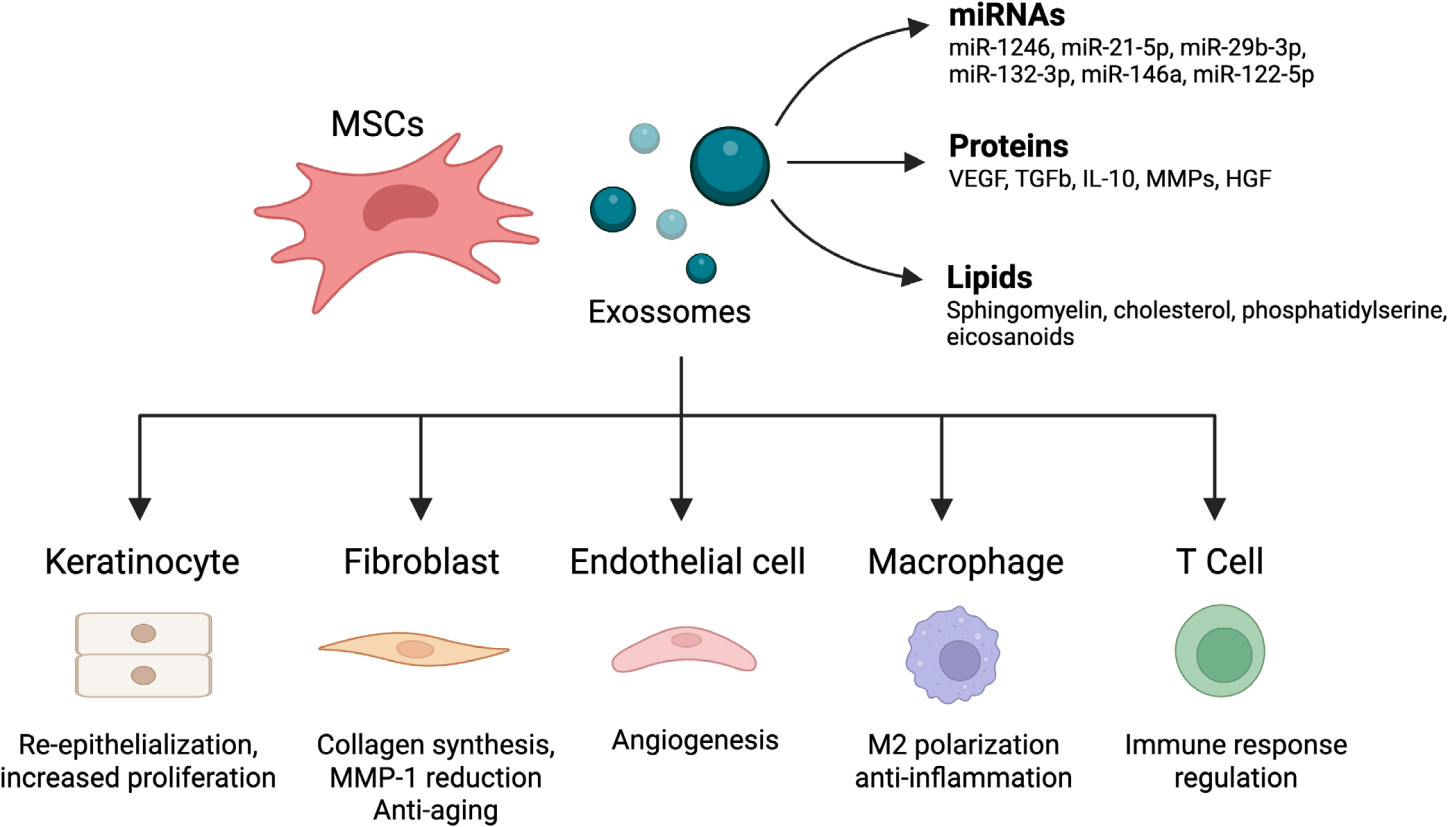
Schematic representation of mesenchymal stem cell (MSC)‐derived exosome components and their mechanistic actions on skin‐resident cells. Exosomes carry microRNAs, proteins, and lipids, which modulate dermal fibroblasts, keratinocytes, endothelial cells, macrophages, and T cells. These interactions contribute to skin regeneration, collagen remodeling, angiogenesis, immunomodulation, and anti‐inflammatory effects relevant to wound healing, photoaging, and inflammatory skin diseases.

Functionally, exosomes modulate immune responses, promote angiogenesis, reduce oxidative stress, and support matrix remodeling and cell proliferation [60, 65, 69]. These actions are particularly relevant in skin regeneration, where rapid inflammation control and stimulation of keratinocyte and fibroblast activity are essential for healing and barrier repair (Figure 4).

Their nanoscale size, lipid bilayer stability, and ability to deliver bioactive cargo across cell membranes make exosomes attractive as acellular therapies for topical or intradermal use in dermatology. These routes favor localized effects, minimize systemic exposure, and align with current clinical approaches to treating inflammatory dermatoses and chronic wounds [22, 23, 60, 69, 70, 71].

## 7 Nucleic Acids and Functional MicroRNAs in MSC‐Derived Exosomes

Transcriptomic and proteomic studies confirm that MSC‐derived exosomes transport diverse nucleic acids, including mRNAs, long non‐coding RNAs (lncRNAs), circular RNAs, and especially miRNAs [61, 65, 72, 73]. These miRNAs are selectively packaged in response to stimuli such as hypoxia, inflammation, and oxidative stress, and modulate gene expression in recipient cells [74, 75, 76, 77].

Several exosomal miRNAs with relevance to skin biology have been identified. miR‐21‐5p and miR‐29b‐3p promote extracellular matrix remodeling, fibroblast migration, dermal repair, and reduce photoaging [78, 79, 80]. miR‐1246, abundant in adipose‐derived exosomes, suppresses MMP‐1 and increases type I procollagen via TGF‐β/Smad and MAPK/AP‐1 modulation, also influencing autophagy through GSK3β inhibition [81, 82]. miR‐132‐3p exerts anti‐inflammatory effects and promotes regeneration by modulating TGF‐β signaling and p300 expression [83]. miR‐146a enhances angiogenesis and delays senescence by targeting Src kinase, VE‐cadherin, and Caveolin‐1 [84]. miR‐122‐5p contributes to hair follicle regeneration by improving dermal thickness and hair bulb size via the TGF‐β1/Smad3 axis [85].

Together, these miRNAs regulate key pathways such as TGF‐β/Smad, MAPK/AP‐1, Wnt/β‐catenin, and NF‐κB, which are crucial for cutaneous immunity, barrier restoration, pigmentation, and adnexal regeneration. Supplementary Table 1 summarizes their molecular targets and functions.

Importantly, the selective enrichment of miRNAs in exosomes offers a mechanistic advantage over the broader, less specific distribution of soluble secretome components. This precision likely enhances the regenerative and immunomodulatory efficacy of exosome‐based therapies, positioning them as refined tools for acellular dermatologic interventions [86].

## 8 Enhancing Therapeutic Efficacy Through MSC Priming

The microenvironment strongly shapes the therapeutic efficacy of MSC‐derived secretome and exosomes during cell expansion. Priming MSCs with stimuli such as inflammatory cytokines, hypoxia, oxidative stress, or 3D culture significantly modifies the composition, potency, and yield of these acellular products [2].

Preconditioning with pro‐inflammatory agents like TNF‐α or TLR4 agonists alters the transcriptome and miRNA cargo of exosomes, influencing their regenerative and immunomodulatory actions. However, some regimens may be detrimental; in ligament injury models, exosomes from inflamed MSCs showed inferior outcomes compared to naive counterparts [87].

Hypoxic priming boosts secretion of angiogenic and neurotrophic factors (e.g., VEGF, HGF) and enhances neutrophil inhibition, potentially broadening anti‐inflammatory applications [88]. Similarly, 3D spheroid cultures mimic in vivo niches, increasing vesicle yield and enhancing regenerative and immunomodulatory activity [89].

Metabolically, primed MSCs package bioactive metabolites into exosomes that drive M2 macrophage polarization and regulatory T cell induction, key for tissue repair and immune tolerance [90]. Enrichment of ECM proteins like fibronectin in primed exosomes may also promote keratinocyte migration and wound remodeling [91].

Despite these advantages, the lack of standardization hampers reproducibility. Key parameters, such as stimulus type, duration, concentration, and culture context, are inconsistently reported, complicating comparisons. Reviews stress that MSC secretome effects are highly context‐dependent, with even minor culture changes altering vesicle content and function [92, 93, 94].

Such variability introduces bias and may underlie inconsistent preclinical outcomes and limited clinical translation. Future studies should clearly define priming protocols, employ indication‐specific potency assays, and include naïve MSC controls to assess the true impact of preconditioning.

MSC priming enhances the therapeutic potential of secretome and exosome products. However, successful translation demands transparent methodology, consistency, and standardized protocols to ensure reproducibility and regulatory alignment [94].

## 9 Engineered Exosomes for Dermatological Applications

Engineered MSCs and their extracellular vesicles, particularly exosomes, are emerging as promising platforms for targeted delivery of therapeutic biomolecules in skin repair and angiogenesis. These vesicles transport proteins, mRNAs, and miRNAs to skin cells, modulating proliferation, migration, angiogenesis, and extracellular matrix remodeling [95, 96, 97, 98, 99].

A key example is the engineering of MSCs to overexpress proteins like Numb, which regulates epithelial stemness and differentiation. Delivered via exosomes, Numb enhances skin regeneration while avoiding the risks associated with live cell therapies [97, 100].

Exosomes can also be enriched with miRNAs—such as miR‐132, miR‐146a‐5p, and miR‐126—that promote wound healing, reduce inflammation, and stimulate vascular growth via gene modulation in target cells [95, 96, 97, 98, 99]. Their retention and targeting have been improved using biomaterial scaffolds and chemoselective immobilization, enhancing in vivo regenerative outcomes [101].

These advances support the development of next‐generation dermatologic therapies with greater specificity and potency. However, challenges remain, including the need for standardized engineering methods, scalable production, and clearer regulatory classification for modified exosomes [97].

Despite these obstacles, current evidence highlights the clinical promise of engineered MSC exosomes in delivering therapeutic proteins and miRNAs for wound healing, photoaging, and vascular regeneration [95, 96, 97, 98, 99, 100, 101, 102, 103, 104, 105].

## 10 MSC Secretome and Exosomes: Applications and Translational Potential in Dermatology

Inflammatory and degenerative skin disorders—such as atopic dermatitis, psoriasis, alopecia areata, vitiligo, and chronic ulcers—are characterized by persistent inflammation, immune dysregulation, and impaired tissue repair [7, 8, 9, 10, 11, 12, 106]. Many present therapeutic challenges, especially in refractory cases [9, 107, 108, 109].

MSC‐derived secretomes and exosomes have attracted interest for their capacity to modulate inflammation, stimulate regeneration, and restore skin homeostasis [4, 5, 6, 20, 21, 52]. As cell‐free therapies, they offer advantages over live cells, including lower immunogenicity, easier storage and handling, scalable production, and compatibility with topical or intradermal routes—particularly relevant in dermatology [4, 6, 7, 20, 21, 23, 106].

Over the past decade, multiple studies have explored their dermatologic applications. Although preclinical results consistently show biological effects across various dermatoses, the strength of evidence remains variable and often constrained by methodological limitations. Below, we review key indications, emphasizing mechanisms, evidence consistency, and translational barriers.

## 11 Wound Healing

Chronic wounds—such as diabetic foot and venous leg ulcers—pose a major clinical challenge due to impaired angiogenesis, persistent inflammation, and defective extracellular matrix remodeling. These lesions often show M1 macrophage dominance, elevated pro‐inflammatory cytokines (e.g., TNF‐α, IL‐1β), and stalled progression through the proliferative and remodeling phases [22, 110]. Inducing a shift toward M2 macrophages is key to resolving inflammation and initiating repair [111, 112].

Wound healing is among dermatology's most studied indications for MSC‐derived secretome and exosomes. Preclinical studies show that these therapies accelerate re‐epithelialization, enhance granulation tissue, and reduce inflammation. In murine models, topical or intradermal application of exosome‐rich formulations improves wound closure and reduces inflammatory infiltrate [79, 113, 114, 115, 116]. These effects are linked to exosomal factors such as VEGF, TGF‐β1, HGF, and pro‐angiogenic miRNAs (e.g., miR‐21, miR‐210), which support matrix remodeling, angiogenesis, fibroblast migration, and keratinocyte proliferation [113, 114, 115].

Though clinical data are limited, early findings are encouraging. Small pilot studies in patients with diabetic or venous ulcers treated with MSC‐conditioned media report improved wound healing, reduced pain, and enhanced tissue quality with minimal adverse effects [117, 118]. These results suggest that secretome‐based therapies may offer valuable adjuncts in refractory cases.

Nonetheless, translational progress faces obstacles. Most animal models simulate acute wounds in otherwise healthy or diabetic mice and fail to capture the chronic complexity of human ulcers [79, 80, 113]. Studies often have small sample sizes and inconsistent reporting of blinding, randomization, and histologic outcomes [113, 114]. Few quantify exosome dose or evaluate biodistribution, limiting cross‐study comparisons [113, 114, 115]. Clinical reports frequently lack control groups, standardized endpoints, or long‐term follow‐up [113, 114, 115]. Variability in MSC source (e.g., adipose, umbilical cord), culture conditions, and secretome isolation further complicate reproducibility [115, 117].

While preclinical evidence strongly supports the regenerative potential of MSC‐derived therapies in wound healing [79, 80, 113, 114, 115], clinical translation is hindered by methodological inconsistencies and the absence of high‐quality randomized trials [117].

## 12 Atopic Dermatitis

Atopic dermatitis (AD) is a chronic inflammatory skin disorder marked by barrier dysfunction, microbiome alterations, and Th2‐dominant immune responses. Conventional therapies, including corticosteroids, calcineurin inhibitors, immunosuppressants, and IL‐4/IL‐13‐targeting biologics, may fail to induce long‐term remission or are limited by side effects [13, 107, 108].

Preclinical data suggest that MSC‐derived exosomes and secretome offer an alternative by modulating inflammation and enhancing barrier repair. In murine models of oxazolone‐induced AD, both systemic and local administration of adipose‐derived MSC (AD‐MSC) exosomes reduced transepidermal water loss, improved stratum corneum hydration, and suppressed proinflammatory cytokines (IL‐4, IL‐5, IL‐13, TNF‐α, IFN‐γ, IL‐17, thymic stromal lymphopoietin [TSLP]) in a dose‐dependent manner [118, 119]. These effects paralleled those of corticosteroids and were accompanied by histological improvements, including reduced epidermal thickness and mast cell infiltration. Treatment also restored ceramide levels and upregulated genes involved in lipid metabolism and barrier integrity [119].

Despite encouraging results, key limitations remain. Most models use acute chemical induction, failing to capture the chronic, relapsing nature of human AD or its complex immunological and microbiome features [118, 119]. Studies vary in administration route (topical vs. subcutaneous), frequency, and exosome dose, hindering cross‐study comparisons [118, 119]. Importantly, no clinical trials to date have evaluated MSC‐derived exosomes in human AD, and comparisons with standard treatments (e.g., corticosteroids, calcineurin inhibitors, dupilumab) are lacking. The effects of these therapies on core clinical outcomes—such as pruritus, SCORing Atopic Dermatitis (SCORAD) score, and quality of life—remain unknown.

Mechanistically, MSC secretome products downregulate Th2 cytokines and restore lipid metabolism while engaging anti‐inflammatory pathways via IL‐10 and TGF‐β signaling [1, 21, 40, 120]. Topical or intradermal administration is advantageous for delivering localized effects with minimal systemic exposure. However, clinical validation is still needed to confirm human safety and efficacy.

## 13 Psoriasis

Psoriasis is a chronic immune‐mediated disease marked by keratinocyte hyperproliferation, abnormal differentiation, and infiltration of Th1/Th17 cells, neutrophils, dendritic cells, and macrophages in the dermis and epidermis. The IL‐23/IL‐17 axis is central: IL‐23 from dendritic cells induces Th17 cells to release IL‐17 and IL‐22, promoting keratinocyte activation and sustaining inflammation [9, 106, 121].

MSCs and their secretome modulate immunity by suppressing Th1/Th17 responses, altering dendritic cell function, and promoting Treg differentiation [122]. These effects involve soluble mediators—TGF‐β1, HGF, PGE2, IDO, and cell contact [1, 17, 35].

However, psoriatic microenvironments may impair endogenous MSCs. MSCs from psoriasis patients (PsO‐MSCs) show increased expression of Th1/Th17 genes and dysfunctional interactions with keratinocytes and immune cells [122, 123, 124].

Recent studies evaluated exosomes from primed MSCs, particularly interferon gamma (IFN‐γ)‐conditioned cells to address this. In imiquimod‐induced psoriasis models, these exosomes reduced skin thickness, erythema, and scaling, while downregulating IL‐17A, TNF‐α, and IFN‐γ [125, 126, 127]. They also increased Tregs and reduced Th17 cells in lesional skin, indicating enhanced immunosuppressive activity [125, 126, 127].

Despite promising preclinical results, challenges remain. The imiquimod model does not fully replicate chronic plaque psoriasis. Few studies directly compare naïve and primed exosomes or identify specific cargo responsible for effects [126, 127]. Clinical trials are lacking, and it is unclear if exosomes can match current biologics targeting IL‐17 or IL‐23 [9, 106, 121]. Optimal delivery routes and durability of response also remain undefined [125, 126, 127].

MSC‐derived exosomes—especially those from primed MSCs—demonstrate potent immunoregulatory effects in preclinical psoriasis models [125, 126, 127]. However, their translation to clinical dermatology requires further mechanistic studies, standardized production, and early‐phase human trials [1, 40].

## 14 Alopecia

Alopecias—including androgenetic alopecia (AGA), alopecia areata (AA), and scarring forms—share key mechanisms: inflammation, follicular injury, and hair cycle disruption. In AA, loss of follicular immune privilege enables T and NK cell–mediated inflammation, suppressing anagen and causing regression. AGA involves dihydrotestosterone (DHT)‐induced TGF‐β/SMAD activation, leading to follicular miniaturization and anagen shortening. Scarring alopecias are driven by chronic inflammation and fibrosis, leading to irreversible follicle loss [23, 24, 128, 129, 130].

MSC‐derived secretome and exosomes may target these mechanisms through anti‐inflammatory, angiogenic, and regenerative effects. These products activate the Wnt/β‐catenin pathway, stimulate VEGF‐mediated perifollicular angiogenesis, and reduce T and NK cell–mediated inflammation [23, 71, 131]. They also promote extracellular matrix remodeling by enhancing collagen and hyaluronic acid synthesis, preserving follicular architecture [132, 133].

Adipose‐derived MSC exosomes modulate TGF‐β1/SMAD3 signaling and deliver miR‐122‐5p, promoting dermal thickening and hair bulb enlargement [85]. In vitro and murine studies show reversal of DHT‐induced suppression of dermal papilla cell (DPC) proliferation, restoration of BMP2, β‐catenin, versican, and cyclin expression, and promotion of anagen entry [85, 131].

Additionally, exosomes reduce oxidative stress, protect against follicular apoptosis, and support immune privilege maintenance [23, 71, 134].

These effects position MSC exosomes and secretome as promising tools across alopecia subtypes, addressing inflammation, vascular dysfunction, and regenerative deficits.

Nonetheless, the therapeutic relevance of these findings is constrained by key limitations. Most studies rely on simplified models—such as DPC monocultures or immunocompromised murine models—that fail to replicate the hormonal complexity of AGA or the autoimmune nature of AA [85, 131]. The current preclinical studies do not assess the immune regulatory effects of exosomes in autoimmune settings, which are critical to understanding their role in non‐scarring alopecias [85, 131]. Furthermore, the optimal route of administration, dosage, and frequency remain undefined, and the durability of the observed regenerative effects is unknown [25, 131].

To date, no human clinical trials have evaluated MSC‐derived exosomes for alopecia, and comparisons with established therapies such as minoxidil, finasteride, or JAK inhibitors are lacking [25]. Therefore, although preclinical evidence points to a regenerative role for MSC‐derived exosomes in hair follicle biology, their translational value remains limited in the absence of rigorous clinical validation.

## 15 Vitiligo

Vitiligo is an autoimmune skin disorder marked by melanocyte destruction and depigmented macules. Its pathogenesis involves genetic susceptibility, oxidative stress, and CD8^+^ T cell–driven immune responses. IFN‐γ and chemokines (CXCL9, CXCL10) promote T cell recruitment and sustain local inflammation [11, 14, 135].

Current therapies aim to suppress autoimmunity and stimulate repigmentation, but results are often incomplete. Topical immunosuppressants offer limited efficacy in extensive disease, while phototherapy requires prolonged use and may raise carcinogenic risk. JAK inhibitors show promise but are costly and carry systemic risks [136, 137, 138, 139].

MSC‐derived exosomes and secretome have emerged as potential alternatives by targeting key pathophysiologic pathways. Preclinical studies suggest dual actions: immune modulation (via CD8^+^ T cell suppression and Treg expansion) and protection of melanocytes from oxidative stress through antioxidant enzymes and microRNAs [14, 56]. Exosomes from 3D‐cultured umbilical cord–derived MSCs inhibit CD8^+^ T cell proliferation and enhance Treg‐mediated immunosuppression while promoting melanocyte viability [56].

Notable exosomal miRNAs include miR‐132‐3p and miR‐125b‐5p, which reduce cytokine production and limit ROS‐induced melanocyte apoptosis [56]. Additionally, the MSC secretome modulates CD8^+^ T cell activity and induces apoptosis in these cells, which are central players in vitiligo pathogenesis [14, 56].

Despite these promising findings, the available evidence remains limited to early‐stage preclinical studies, often relying on surrogate markers such as tyrosinase activity or antioxidant gene expression [56]. No studies have demonstrated durable repigmentation or lesion clearance in human skin. There are no clinical trials of exosome‐based therapies in vitiligo [14, 25, 56]. Furthermore, the complex immunopathogenesis of vitiligo—including the role of Langerhans cells and tissue‐resident memory T cells—remains underexplored in this context [14]. The extent to which systemically administered exosomes can achieve targeted cutaneous effects without inducing off‐target immunosuppression is also unclear, as biodistribution studies in dermatologic applications are lacking [56].

While MSC‐derived exosomes and secretome show potential by targeting inflammation and oxidative injury [14, 56], translation to clinical care requires disease‐relevant models, standardized endpoints, and human trials.

## 16 Photoaging

Photoaging arises from cumulative ultraviolet (UV) exposure, leading to collagen degradation, extracellular matrix (ECM) disorganization, oxidative stress, and upregulation of matrix metalloproteinases (MMPs) [118]. These alterations manifest clinically as wrinkles, dyspigmentation, loss of elasticity, and dermal thinning.

MSC‐derived secretome and exosomes have shown promise in mitigating these effects through regulation of fibroblast function, modulation of inflammatory responses, and restoration of ECM architecture. In ultraviolet B (UVB)‐induced photoaging models, exosomes from adipose‐derived MSCs enriched with miR‐1246 suppressed MMP‐1, enhanced type I collagen synthesis, and attenuated oxidative damage by modulating TGF‐β/Smad, MAPK/AP‐1, GSK3β, and autophagy pathways [79, 140]. Likewise, miR‐29b‐3p, delivered via bone marrow–derived exosomes, inhibited MMP expression and prevented collagen degradation, supporting ECM integrity and dermal resilience [140].

These findings support the use of MSC‐derived exosomes as topicals or intradermal agents in aesthetic dermatology, potentially enhancing outcomes and shortening recovery in procedures such as laser resurfacing and microneedling [79, 81, 82, 140].

However, most evidence stems from preclinical models using young animals or isolated fibroblast cultures, which do not fully recapitulate the cumulative photodamage and structural senescence of aged human skin [79, 82, 140]. Moreover, outcomes like wrinkle severity, skin elasticity, and dermal thickness are rarely assessed using standardized, blinded methods, limiting the robustness of comparisons [79, 82].

To date, no randomized clinical trials have evaluated MSC‐derived exosomes in aesthetic indications, and few studies compare their efficacy against established treatments such as retinoids, microneedling, or energy‐based devices [140, 141]. Safety data remain scarce, particularly regarding potential pro‐fibrotic effects, immunogenicity, and biodistribution in cutaneous tissue.

While MSC‐derived secretome and exosomes exert biological actions consistent with anti‐aging goals, their integration into aesthetic dermatology will require validation through rigorously designed human trials employing standardized and objective outcome measures.

## 17 Rare and Emerging Indications

Beyond common inflammatory and degenerative dermatoses, MSC‐derived secretome and exosomes are being explored for rare and complex skin disorders such as hidradenitis suppurativa (HS) and epidermolysis bullosa (EB). Although still experimental, these investigations highlight the expanding therapeutic potential of MSC‐based approaches.

Hidradenitis suppurativa is a chronic, relapsing inflammatory condition marked by nodules, abscesses, and sinus tracts in intertriginous areas. Its pathogenesis involves follicular occlusion, dysregulated cytokines—especially TNF‐α and IL‐1β—and persistent immune activation, contributing to chronic inflammation and defective wound healing [142, 143]. Preclinical evidence suggests that MSC‐derived secretome may modulate these pathways. In vitro, primed MSCs suppress the proliferation of peripheral blood mononuclear cells and downregulate IL‐6, IL‐9, IL‐17A, and IFN‐γ [144]. These effects are amplified by preactivation with TNF‐α and IFN‐γ, enhancing immunosuppressive activity, M2 macrophage polarization, and NF‐κB inhibition [144]. While no in vivo or clinical trials have assessed secretome or exosomes in HS yet, this mechanistic rationale supports further investigation, particularly in refractory cases.

Epidermolysis bullosa, notably its recessive dystrophic subtype (RDEB), is a severe inherited blistering disorder caused by mutations in *COL7A1*, leading to loss of type VII collagen and dermoepidermal anchoring fibrils [145, 146, 147]. This results in extreme skin fragility, trauma‐induced blistering, chronic ulcers, and fibrosis. In *Col7a1*‐deficient mice, wound healing is further impaired by defective granulation tissue formation, reepithelialization, and cell migration [147]. MSC‐derived extracellular vesicles have demonstrated the ability to deliver *COL7A1* mRNA or protein to patient‐derived fibroblasts, partially restoring collagen VII expression and dermoepidermal adhesion [148]. In parallel, the MSC secretome promotes angiogenesis, modulates inflammation, and enhances reepithelialization—features that may complement gene therapy or act as supportive treatments [149].

Although current studies in HS and EB remain limited to preclinical models, they underscore the broader applicability of MSC‐derived products in high‐need dermatologic conditions. Robust preclinical validation and early‐phase clinical trials will be essential to evaluate safety, delivery strategies, and therapeutic efficacy.

## 18 Safety Concerns: Could MSC‐Derived Exosomes Promote Skin Cancer?

The use of MSC‐derived secretome and exosomes in dermatologic therapies raises legitimate safety concerns, especially in chronically inflamed or photo‐damaged skin settings already linked to higher cancer risk. Their oncogenic potential lies in the anti‐apoptotic, proangiogenic, and immunomodulatory properties of MSC‐secreted factors. While beneficial for repair, these can also foster a tumor‐promoting microenvironment, particularly in areas with subclinical or pre‐existing lesions [150, 151].

Experimental models show that MSC‐derived products may enhance melanoma growth, invasiveness, and resistance to treatment via paracrine signaling. The secretome, rich in cytokines, growth factors, and exosomes, can influence surrounding cells—including malignant ones. Depending on context, exosomal microRNAs may exert tumor‐suppressive or tumor‐promoting effects, affecting both melanoma and non‐melanoma cancers [150, 151].

Though standard toxicology studies have not shown direct carcinogenicity, they may miss subtle tumor‐promoting effects, particularly in high‐risk skin environments [152]. Exosomal stimulation can produce unpredictable outcomes in photo‐damaged or inflamed skin, which is already structurally and genetically altered.

Despite being acellular, these therapies modulate oncogenic pathways such as immunosuppression, angiogenesis, and apoptosis inhibition, which may inadvertently support tumor persistence [32, 153]. These context‐dependent effects underscore the need for robust preclinical testing and careful clinical use, especially in patients with increased cancer susceptibility.

While MSC‐derived exosomes show regenerative potential, their long‐term safety remains unclear. Most studies lack prolonged follow‐up to detect delayed tumorigenic effects. Clinical use should include rigorous safety monitoring. Regulatory frameworks must incorporate cancer risk assessment, particularly for repeated or localized skin applications. Continued research into their dual regenerative and oncogenic roles is key for safe clinical translation [32, 150, 151, 152, 153].

## 19 Challenges in Clinical Translation

Despite promising preclinical results and rising clinical interest, several barriers hinder the translation of MSC‐derived secretome and exosomes into dermatology. Key challenges include product heterogeneity, methodological inconsistency, unclear pharmacokinetics, and unresolved safety and regulatory issues.

A major limitation is the lack of standardization in MSC isolation, expansion, and conditioning. Variables such as tissue source (e.g., bone marrow, adipose, umbilical cord), donor age, passage number, priming (e.g., hypoxia, cytokines), and culture method (2D vs. 3D) significantly affect the composition and bioactivity of secretome and EVs [2, 20, 40, 59]. These heterogeneities alter key mediators like VEGF, IL‐10, TGF‐β, and miRNAs, leading to batch variability and limiting reproducibility, scalability, and regulatory compliance [2, 40, 59, 154].

EV isolation and quantification methods also vary—ultracentrifugation, size‐exclusion chromatography, and polymer‐based precipitation yield vesicles with differing purity and cargo. Lack of consensus on EV nomenclature and classification complicates comparisons. Most studies report protein, particle count, or RNA mass concentrations, but dosing, frequency, and delivery routes remain unstandardized in dermatology [2, 40, 59].

Safety is another major concern. Although exosomes are less immunogenic than whole MSCs, their biodistribution and clearance after topical or intradermal use remain poorly understood. They may be internalized by off‐target cells, posing risks in immunocompromised or cancer‐prone individuals [97]. Residual DNA, cytokines, or pro‐inflammatory signals may persist, with unknown long‐term effects. As previously noted, MSC secretome may influence the tumor microenvironment and promote oncogenesis via angiogenic and immunosuppressive pathways [16, 35, 155].

Senescence adds further variability. Senescent MSCs acquire an SASP, which is marked by elevated inflammatory cytokines and reduced regenerative potential [156, 157]. Strategies like hypoxic preconditioning and 3D culture aim to reduce these effects and improve consistency [158].

No MSC‐derived secretome or exosome product has yet received FDA or EMA approval for dermatologic use. While regulatory frameworks are evolving, current requirements—including safety testing, potency validation, and GMP manufacturing—pose substantial barriers for academic and early‐stage developers [97, 159].

## 20 Conclusion

MSC‐derived secretomes and exosomes offer a promising cell‐free therapeutic approach for various dermatological conditions. These acellular products modulate inflammation, stimulate tissue regeneration, and promote immune homeostasis in inflammatory and degenerative skin disorders by delivering bioactive molecules such as cytokines, growth factors, and regulatory RNAs.

Preclinical studies consistently support their efficacy in models of atopic dermatitis, psoriasis, alopecia, vitiligo, chronic wounds, and photoaging. Early‐phase clinical data, though limited, indicate favorable safety and feasibility for topical and intradermal application, with encouraging results in treatment‐resistant cases.

However, clinical translation remains constrained by unresolved challenges, including product heterogeneity, lack of standardized dosing, limited pharmacokinetic data, and regulatory uncertainty. The absence of approved therapies underscores the need for harmonized protocols, mechanistic validation, and well‐designed clinical trials.

As scientific knowledge and regulatory pathways evolve, MSC‐derived secretome and exosomes may emerge as a novel class of biologics capable of filling therapeutic gaps, especially in patients unresponsive to conventional treatments. With interdisciplinary collaboration and methodological rigor, these acellular strategies hold potential to advance from experimental models to routine dermatologic care.

## Conflicts of Interest

The authors declare no conflicts of interest.

## Supporting information


**Data S1:** Supplementary Material.

## Data Availability

Data sharing not applicable to this article as no datasets were generated or analysed during the current study.
