# Supplementary material for: MSC‐Derived Secretome and Exosomes in Dermatology: Mechanisms, Therapeutic Opportunities, and Scientific Challenges—A Narrative Review
Source: Int J Dermatol. 2025 Aug 1;65(2):257–72. doi: 10.1111/ijd.17982 (PMC12783422; doi:10.1111/ijd.17982)
Supplement: Supplementary file 1 — Data S1: Supplementary Material. [file IJD-65-257-s001.docx]

**Supplementary Figure 1.** Exosome Morphology.


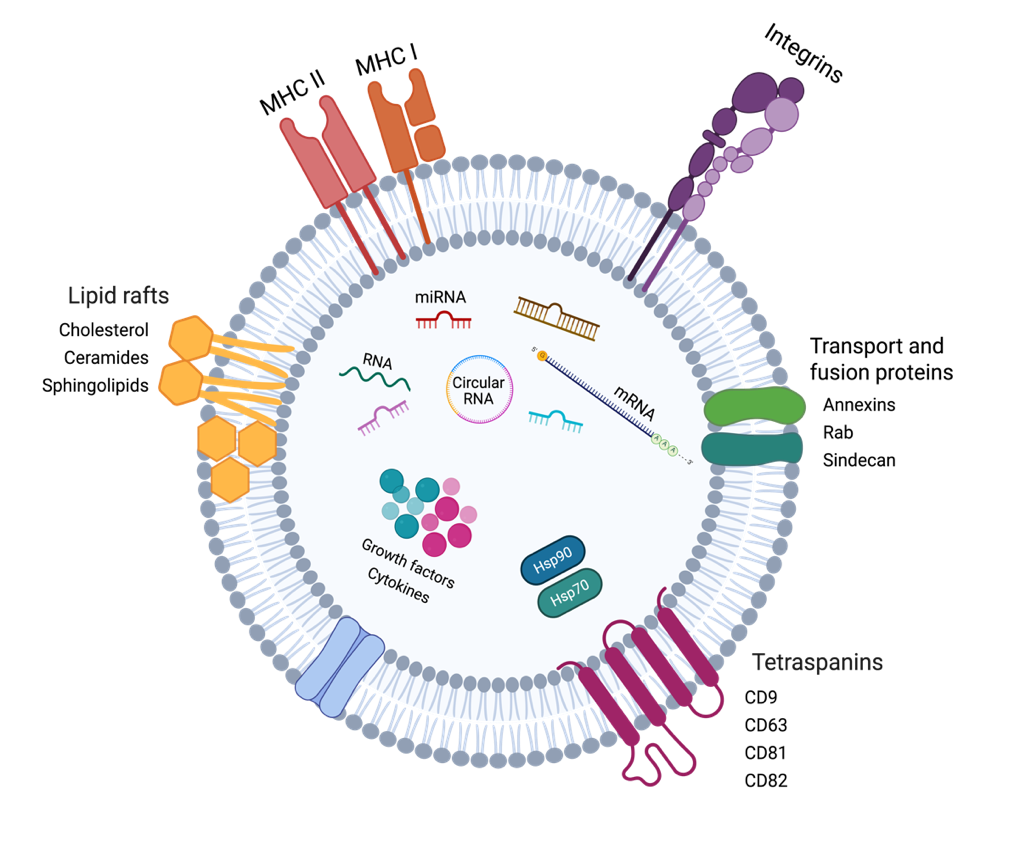


Created in BioRender. Cestari, M. (2025) https://BioRender.com/dtqmke4

| **Supplementary Table 1.** Summary of miRNAs molecular targets and functions. | | | | |
| --- | --- | --- | --- | --- |
|  | miRNA | Pathways  or Targets | Effects | Ref. |
| **ADMSC** | miR-34a-5p  miR-124-3p  miR-146a-5p | RG1  CD206  TSG-6  TGF-β1 | Polarization of macrophages toward the M2 phenotype | ^77^ |
|  | miR-125a | DLL4 | Endothelial cell proliferation | ^163^ |
|  | miR-486-5p | Sp5  CCND2 | **Proliferation and migration of fibroblasts and human dermal microvascular endothelial cells (HMECs)**  **Increased angiogenesis of human dermal microvascular endothelial cells (HMECs)** | ^164^ |
|  | miR-10b | PEA15 | **Proliferation and migration of** keratinocytes  Decreased apoptosis | ^165^ |
|  | miR-19b | CCL1  TGF-β | **Proliferation and migration of** keratinocytes and fibroblasts  Decreased apoptosis | ^166^ |
|  | miR-141-3p | TGF-β2  Smad2/3 | Decreased myofibroblasts action  Modulating activity in hypertrophic scar formation | ^167^ |
|  | miR-29a | TGF-β2  Smad3 | Decreases fibroblast migration and proliferation  Reduction of collagen deposition and fibrosis in the extracellular matrix (ECM) | ^80^ |
|  | miR-181a | Sirtuin1 | Reduced differentiation into myofibroblasts  Decreased collagen deposition | ^168^ |
|  | miR-192-5P | IL-17RA  Smad | Decreased fibroblast migration and proliferation  Reduced differentiation into myofibroblasts  Decreased collagen deposition | ^169^ |
|  | miR-146a | Src kinase  VE-cadherin  Caveolin-1 | Increased angiogenesis  Decreased cellular senescence | ^84^ |
|  | miR-22 ↓ | Wnt/βcatenin  TNF-α | **Capillary growth**  **Capillary regeneration**  **Increased dermal thickness**  **Increased migration and proliferation of dermal papilla cells** | ^170^ |
|  | miR-122-5p | TGF-β1  Smad3 | I**ncreased hair bulb size**  **Increased dermal thickness** | ^171^ |
| **BMSC** | miR-223 | Pknox1 | Macrophage polarization toward the M2 phenotype | ^113^ |
|  | miR-221-3p | AKT  eNOS | **Increased VEGF expression**  **Granulation tissue formation** | ^114^ |
|  | miR-93-3p | APAF1 | **Keratinocyte proliferation and migration**  **Decreased apoptosis** | ^115^ |
|  | miR-21-5p | SPRY2 | **Decreased fibroblast migration and proliferation**  **Reduced collagen deposition in the ECM** | ^79^ |
|  | miR-29b-3p | MMP-2  TGF-β  Smad  MAPK/AP-1 | **Increased dermal fibroblast migration**  **Reduced photoaging**  **Decreased MMP levels**  **Increased procollagen** | ^141,172^ |
| **UCMSC** | miR-let-7b | TLR4  NF-κB  STAT3  AKT | **Macrophage polarization toward the M2 phenotype**  **Reduced production of pro-inflammatory cytokines** | ^173^ |
|  | miR-181c | TLR4  NF-κB  P65 | **Reduced production of pro-inflammatory cytokines** | ^174^ |
|  | miR-125b | TP53INP1 | **Endothelial cell proliferation and migration**  **Decreased apoptosis** | ^175^ |
|  | miR-21,  miR-23a,  miR-125b,  miR-145 | TGF-β2  Smad2 | **Reduced differentiation into myofibroblasts**  **Decreased collagen deposition** | ^176^ |
|  | miR-493-3p | TNF-α  NF-κB | **Fibroblast proliferation and migration**  **Increased procollagen**  **Reduced oxidative stress**  **Decreased cellular senescence** | ^177^ |
|  | miR-196a-5p | NF-κB | **Fibroblast proliferation and migration**  **Increased procollagen**  **Reduced oxidative stress**  **Decreased cellular senescence** | ^177^ |
